# Spatial and chromatic properties of numerosity estimation in isolation and context

**DOI:** 10.1371/journal.pone.0274564

**Published:** 2022-09-15

**Authors:** Elena Gheorghiu, Dirk Goldschmitt

**Affiliations:** University of Stirling, Department of Psychology, Stirling, Scotland, United Kingdom; French National Center for Scientific Research (CNRS) & University of Lyon, FRANCE

## Abstract

Numerosity estimation around the subitizing range is facilitated by a shape-template matching process and shape-coding mechanisms are selective to visual features such as colour and luminance contrast polarity. Objects in natural scenes are often embedded within other objects or textured surfaces. Numerosity estimation is improved when objects are grouped into small clusters of the same colour, a phenomenon termed groupitizing, which is thought to leverage on the subitizing system. Here we investigate whether numerosity mechanisms around the subitizing range are selective to colour, luminance contrast polarity and orientation, and how spatial organisation of context and target elements modulates target numerosity estimation. Stimuli consisted of a small number (3-to-6) of target elements presented either in isolation or embedded within context elements. To examine selectivity to colour, luminance polarity and orientation, we compared target-only conditions in which all elements were either the same or different along one of these feature dimensions. We found comparable performance in the same and different feature conditions, revealing that subitizing mechanism do not depend on ‘on-off’ luminance-polarity, colour or orientation channel interactions. We also measured the effect of varying spatial organisation of (i) context, by arranging the elements either in a grid, mirror-symmetric, translation-symmetric or random; (ii) target, by placing the elements either mirror-symmetric, on the vertices of simple shapes or random. Our results indicate higher accuracy and lower RTs in the grid compared to all other context types, with mirror symmetric, translation and random arrangements having comparable effects on target numerosity. We also found improved performance with shape-target followed by symmetric and random target arrangements in the absence and presence of context. These findings indicate that numerosity mechanisms around the subitizing range are not selective to colour, luminance polarity and orientation, and that symmetric, translation and random contexts organisations inhibit target-numerosity encoding stronger than regular/grid context.

## 1. Introduction

Extracting the number of objects in natural scenes at a glance is an integral part of everyday tasks. Humans can rapidly and accurately estimate small number of objects (up to between 4 to 7) without relying on cognitive processes such as counting, which are not effective at short viewing times [1]. This process, known as subitizing [2–5], is quick and highly accurate when the number of objects is less than four, while numerosities between 5 and 7 (see [4–12] for a subitizing limit of up to 7) are estimated quickly but with lower accuracy [13]. The subitizing limit, as defined by Kaufman and Lord [5], is the discontinuity point in the distribution of reaction times or accuracy. Many studies identified a subitizing limit between 4 and up to 7 items depending on the type of stimuli and procedures employed, and on the methods used for calculating the limit for the subitizing range, that is, bilinear fit (also known as piecewise regression or ‘broken stick‘ fit) versus sigmoidal (S-shaped) fit [6,7,9–19]. Behavioural and electrophysiological support for the idea of distinct neural processes involved in small (up to 4) and large numerosity estimation comes from numerosity perception studies in adults [13,20] and children [21]. For larger numbers, beyond the subitizing limit, numerosity perception is sub-served by mechanisms activated by different ranges of numerosity (for a review see [22,23]). Other studies however found no evidence of a significant discontinuity in reaction times with increasing numerosity beyond the subitizing range [24], suggesting that the mental effort involved in numerosity estimation increases with each item added to the stimulus, both within and beyond the subitizing range, with no implication of two (or more) distinct processes.

Numerosity perception around the subitizing range has been found to be inherently related to geometric cues in the formation of an object’s shape as demonstrated with dice dot-patterns [9,19,25], and simple shapes such as triangle, square, pentagon and hexagon [26]. Global shape processing such as radial-frequency (RF) shape patterns (i.e., shapes created by sinusoidally modulating a circle’s radius with the number of full cycles of modulation per 2π radians being the RF number: RF3-triangle, RF4-square, RF5-pentagon, etc.) is thought to depend upon global pooling of information about the peaks and troughs (i.e., points of maximum curvature or vertices/ corners) [27–29]. This global processing limit extends between RF5 (pentagon) and up to RF7 (heptagon) [27,28], while higher frequency RFs patterns rely on local processing of orientation and position information. Thus, global shape processing is limited by the number of shape vertices [27] which is comparable with the subitizing limit [4–12]. Recently, Gheorghiu and Dering [26] showed that spatial configuration and its complexity (i.e., the number of shape vertices) affect numerosity estimation, with better performance for shape compared to random arrangements and for simple (triangle) compared to complex (hexagon) shapes. Their findings indicate that shape coding *precedes* numerosity estimation, which implies that numerosity estimation around the subitizing range is *facilitated* by a shape-template matching process which takes into account the relationship between points of maximum curvature (or vertices). This idea received further support from studies showing that sensitivity to visual form (Glass patterns) can predict numerical abilities [30]. Previous studies of shape processing have shown that an inconsistency in local features, such as luminance polarity and orientation of elements that make up a shape, strongly disrupts the encoding of global shape for long straight lines [31], curves [32,33], radial-frequency patterns [34,35] and Glass patterns [36]. Shape coding mechanisms have been found to be selective (or tuned) to features such as luminance contrast polarity [32], colour [37,38] and local orientation [31,39]. This raises the question as to whether numerosity estimation around the subitizing range, which is facilitated by a global shape-template matching process [26], is also disrupted by differences in the visual features of the elements.

In addition, objects (or a group of objects) in natural scenes rarely occur in isolation but are often embedded within other irrelevant objects or textured surfaces. Although it is known that salient visual features such as mirror-symmetry and regularity can bias our perception of scene content [40–42], the extent to which spatial regularities of *context* elements affect numerosity estimation of a group of objects (*target*) remains unknown.

In this communication, we consider whether numerosity estimation around the subitizing range is selective to colour, luminance polarity and orientation of elements, and whether spatial organisation of context and of target elements modulates target numerosity perception.

While some studies make an implicit assumption that numerosity perception is invariant to low level features of elements such as luminance contrast polarity [43–45], others have shown that differences in luminance contrast polarity [46] and orientation [47] of elements affect numerosity perception, suggesting that numerosity estimation mechanisms are selective to these features. Using relatively large numerosity displays (128 elements) made of either single polarity (all white or all black) or different polarity (white and black) elements, Tibber et al [46] found a small increase in sensitivity thresholds for numerosity estimation for same compared to different polarity conditions, suggesting that luminance contrast polarity consistency can facilitate numerosity perception.

Orientation consistency (or coherence) and connectedness (or collinearity) have been also found to affect numerosity estimation above the subitizing range [47]. DeWind et al [47] reported that stimuli made of similarly oriented items (8–32 oriented Gabors) were perceived as more numerous than randomly oriented items, an effect termed ‘coherence illusion’. Specifically, they found that aligning the orientations of items increased their perceived numerosity, whereas increasing the orientation variance of the stimuli decreased perceived numerosity. On the other hand, adaptation studies showed that numerosity aftereffects induced by vertically-oriented adaptor elements in either vertical or horizontal-oriented test elements were similar in magnitude [48], suggesting that numerosity mechanisms are not selective for orientation. Hence, the extent to which numerosity mechanisms are selective and/or sensitive to orientation distribution of the elements remains unclear.

The literature is equivocal on the issue of whether there exist colour selective channels for numerosity estimation. One single recent study has examined whether numerosity mechanisms mechanism above the subitizing range are tuned to colour [49]. Using higher numerosity adapting patterns (48 elements), Grasso et al [49] reported that numerosity aftereffects induced in lower-numerosity test patterns (12–48 elements) were reduced when the adaptor and test were the same (or perceptually matched in) colour compared to being different in colour. These authors found a 25% underestimation of test numerosity for matched colours, while different colours had little or no effect on the magnitude of numerosity aftereffect, thus arguing that numerosity mechanisms above the subitizing range are colour-selective. However, the adaptor and test patterns used in [49] (and also in [48]) differed not only in terms of their numerosity but also in elements’ (or texture) density. Hence, the sense of number and sense of density are intertwined in such stimuli as it has been demonstrated by several studies [50–52]. Given that texture-density aftereffects were found to be selective to colour [53,54], it remains unclear to what extent numerosity mechanisms are selective to colour.

Other studies investigating the role of colour in numerosity perception have been focused on the effect of colour grouping (or colour similarity) on numerosity estimation in stimuli of relatively large numerosities, above the subitizing range, that were grouped into small clusters of elements of the same colour [55–60]. While some studies found an underestimation of numerosity when neighbouring elements in an array were of the same colour (i.e., colour duplicates) compared to random colours [58], an effect which could not be explained by increased attention to colour, others reported no underestimation effect [57,59] or an improved performance when elements were grouped by colour [55]. Anobile et al. [55] showed that when arrays of elements are grouped into small clusters (of no more than 5–6 elements) of the same colour, numerosity estimation is more rapid and accurate as compared to when the elements in the clusters were of different colours. This effect, termed ‘groupitizing’ [61–63], is thought to rely on the recruitment of the subitizing system [55,56,61]. Therefore, these findings showing either improved [55] or poorer [58] performance in colour grouped displays indirectly suggest that numerosity estimation within the subitizing range might be selective or tuned to colour (i.e., there are separate numerosity processing channels for each colour, that is, a red numerosity channel, green numerosity channel and so on). However, to our knowledge, there are no studies that directly examined whether numerosity perception mechanisms around the subitizing range are selective to colour, luminance polarity and orientation. On the grounds of the above-mentioned studies, we might expect numerosity estimation mechanisms around the subitizing range to be, at least to some extent, selective to these features.

Many studies use colour and/or luminance contrast polarity to isolate or individuate a spatially clustered subset of relevant elements (target) from irrelevant distractor elements (or context), an effect often referred to as ‘individuation’ [64–68], to examine how the presence of distractor items affects the behavioural and electrophysiological responses to target numerosity estimation within and above the subitizing range [6,66,69,70]. While some studies have reported that increasing the number of distractor elements can lead to decreased performance for target numerosity estimation, with the rate of decline being somewhat different below and above 4 elements [6,65], others found a similar rate of decline in performance across the entire numerosity range [71]. However, to our knowledge there are no studies that examined how the spatial organisation of context (or distractor elements) and of target elements affect target numerosity perception.

With regard to spatial organisation of elements, it is known that mirror-symmetry and regularity of pattern elements can bias our perception of large numerosities, with symmetric patterns appearing to have fewer elements [42] and regular patterns being perceived as more numerous than random patterns [40,41]. Although recent studies demonstrated that spatially organising target items into simple shape configurations such as simple geometric shapes [26] or dice-like patterns [9,19,25] can facilitate numerosity perception in the absence of context, it remains unknown how the presence of context and the spatial organisation of both context and target elements affect target numerosity estimation.

In this study, we determine whether numerosity mechanisms around the subitizing range are selective to elements’ colour (Experiment 1), luminance polarity (Experiment 2) and orientation (Experiment 3) and if they are, then establish whether these selectivities are derived from separate feature-selective shape channels (e.g., red-shape channel, blue-shape channel, green-shape channel, etc.) or arise at a stage where numerosity is directly encoded (i.e., beyond the shape-facilitation level). To do this, we compared accuracy and reaction times for stimuli made of a small number of elements (3, 4, 5 or 6) positioned either on the vertices of simple geometric shapes (equilateral triangle, square, pentagon and hexagon) or random, and defined along either the same (all same colour; all white or all black) or different (mixed colours; white and black) feature dimensions. To test for orientation selectivity (Experiment 3), the elements were oriented either collinear or orthogonal to the contour-shape path. If consistency of features can facilitate numerosity estimation in a similar way to shape perception [31–35], and as indirectly suggested by groupitizing studies [55,58], we expect higher accuracy and faster reaction times in the *same* compared to *different* feature conditions, suggesting that numerosity estimation mechanisms around the subitizing range are selective to colour/ luminance polarity/ orientation. If these selectivities occur only when the elements are arranged in simple shape configurations but not random, then these selectivities are inherited from feature-selective shape channels (e.g., red-shape channel, blue-shape channel, etc.). If better performance for same compared to different feature conditions occurs for *both shape and random* configurations, then it suggests that feature selectivity arises directly at the stage where numerosity is encoded. Conversely, if same and different feature conditions produce comparable performance irrespective of spatial arrangement (shape vs. random) then it implies that numerosity mechanisms are not selective to colour/ luminance polarity and orientation.

Finally, in Experiment 4 we examined how spatial regularities of context elements (e.g., mirror symmetry, translation symmetry and regularity/grid) and of target elements (shape and mirror symmetry) modulate target numerosity estimation. Previously we showed that when target elements are presented in isolation (no context), performance was better when elements were placed in shape compared to random configurations [26]. Hence, in the presence of context, we expect that spatial regularities of context will result in reduced accuracy of target numerosity estimation, and that shape and symmetric target arrangements will produce higher accuracy compared to random target arrangements.

## 2. Methods

### 2.1. Participants

A total of 175 observers between 18 to 60 years of age, who were naive in regard to the experiment aims participated in this study: 46 in Experiment 1 (mean age: 24, age range:18–44, with 36 participants between 18–30 years), 39 in Experiment 2 (mean age: 22, age range: 18–35, with 33 participants between 18–30 years), 43 in Experiment 3 (with 35 participants between 18–30 years and 8 participants between 31–50 years), and 47 in Experiment 4 (with 34 participants between 18–30 years, 9 participants between 31–50 years, and 4 participants over 51 years). All observers had normal or corrected to normal visual acuity. Observers gave their written informed consent prior to participating and were all treated in accordance with the Declaration of Helsinki (2008, version 6). All research procedures were approved by the University of Stirling Ethics Committee.

### 2.2. Stimuli

The stimuli were generated in Matlab and presented on a Sony Trinitron monitor with a 1024 x 768 spatial resolution and a refresh rate of 120 Hz (Experiment 1 and 2). The R (red), G (green) and B (blue) outputs of the monitor were gamma-corrected after calibration with an Optical OP200E photometer. All stimuli were presented in the centre of the monitor on a uniform mid-grey background with mean luminance of 65.5 cd/m^2^. Viewing distance was 100 cm. For Experiments 3 and 4, stimuli were presented using Testable (https://www.testable.org/) on individual participants computer monitors.

In Experiments 1–3, stimuli consisted of a small number of elements, either 3, 4, 5 or 6 presented in the centre of the monitor, within a circular area of 8 deg of visual angle diameter. In different experiments, we varied the visual features defining the elements and their spatial arrangement or configuration. We did not use stimuli made of 1 and 2 elements as their arrangement cannot dissociated between shape and random configurations (e.g., any two randomly placed elements will always make a straight line) and/or between any simple geometric shapes. The elements were either chromatic (Experiment 1) or luminance-defined (Experiment 2) Gaussian blobs with a standard deviation of 0.08 deg, a Gaussian size standard deviation factor of 5 and a contrast of 0.9. The chromatic Gaussian elements were non-isoluminant. In Experiment 3, the elements were odd-symmetric (d.c. balanced) achromatic Gabor patches with a spatial bandwidth of 1.5 octaves, centre luminance spatial frequency of 4 c/deg, and a contrast of 0.9.

**Fig 1:**
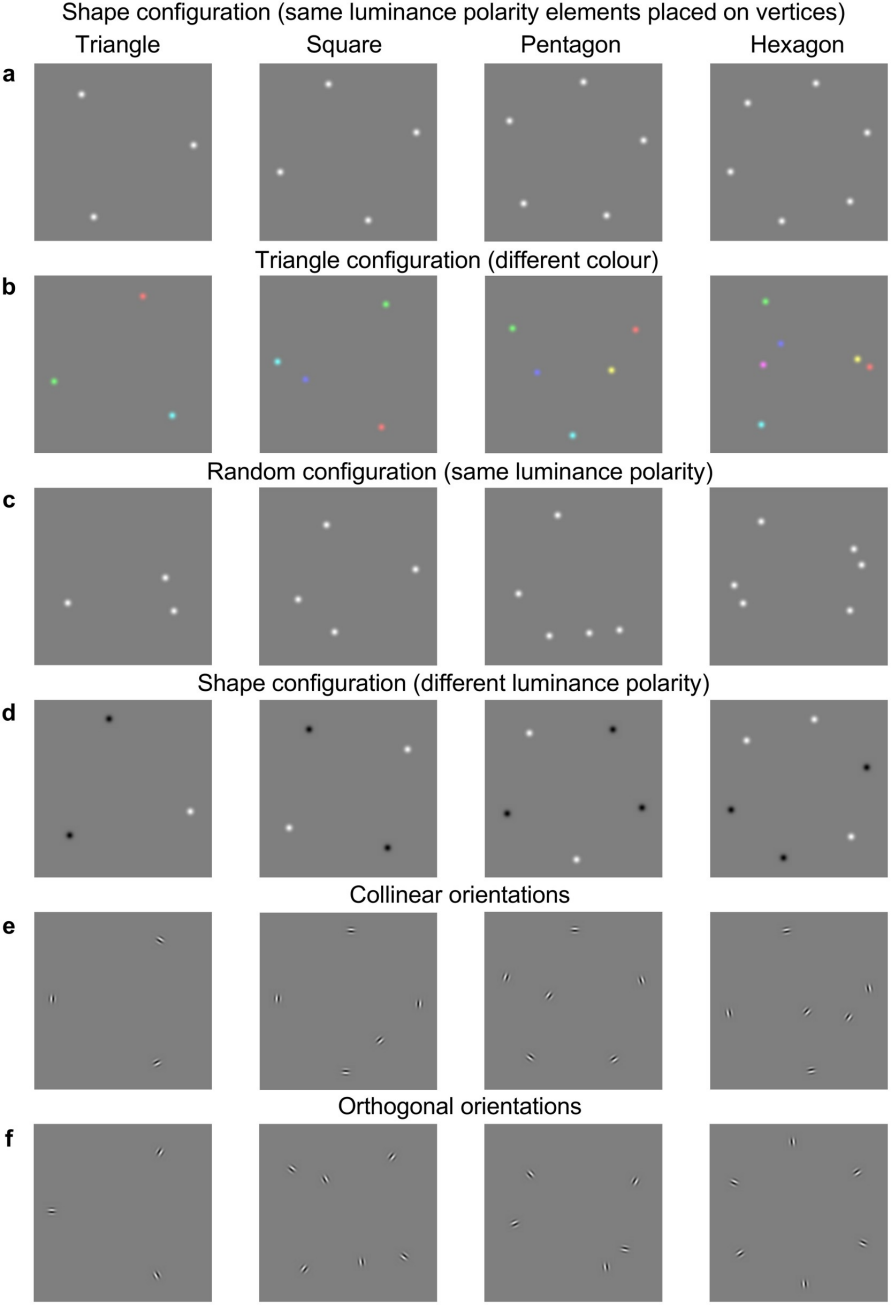
Example stimuli used in Experiments 1–3. Stimuli consisted of a small number of elements (3, 4, 5 or 6) positioned either **(a)** on the vertices of simple shapes, e.g., equilateral triangle, square, pentagon and hexagon or **(c)** random, e.g., elements placed randomly anywhere within and including the virtual contour-path except on the vertices. **(b)** Equilateral triangle sampled by either 3, 4, 5 or 6 elements, with three elements always positioned on the vertices and the remaining elements placed randomly anywhere within and including the virtual contour-path. **(d)** Stimuli made of different luminance-polarity elements (white and black). Elements had either same (a,c) or different (d) luminance-polarity (Experiment 2), and either same or different (b) colour (Experiment 1). **(e,f)** Experiment 3 stimuli made of Gabor elements oriented either (e) collinear or (f) orthogonal to the virtual path of simple contour shapes. For all experiments, the orientation of each virtual shape configuration was randomized from trial to trial.

To examine the effect of colour (Experiment 1), luminance polarity (Experiment 2) and orientation (Experiment 3), we used stimuli in which the elements were either the same or different along a particular dimension e.g., same (all white or all black; Fig 1a) vs different (white and black; Fig 1d) luminance polarity and same colour (as in Fig 1a but with all blobs being of one colour which was randomly selected from the available colours, e.g., all red, all green, all blue, etc.) vs. different colours (Fig 1b). In Experiment 3, the Gabor patches were oriented either collinear or orthogonal to the virtual path of simple contour shapes (Fig 1e and 1f).

To examine the effect of spatial configuration, the elements were positioned either on vertices (points of maximum curvature) of simple geometric shapes (equilateral triangle, square, pentagon and hexagon; Fig 1a), or random (Fig 1c). For the *on-vertices* (or *shape) condition*, an equilateral triangle was sampled by either 3, 4, 5, or 6 elements with 3 elements always placed on the vertices and the remaining elements placed randomly anywhere on the virtual contour path and inside the shape area (Fig 1b); the square shape was sampled by either 4, 5, or 6 elements, with 4 elements placed on the vertices and the remaining elements placed randomly on the virtual contour path and inside the shape area, and so on. The orientation of each virtual shape configuration was randomized from trial to trial. For the *random condition* (Fig 1c), the elements were placed anywhere inside the shape area and on the virtual contour path, *except* on its vertices. To avoid spatial overlap, the minimum distance between the elements was set to be at least twice their size. Given the number of elements (3, 4, 5, 6), spatial configuration (on-vertices/shape vs. random), shape complexity (equilateral triangle, square, pentagon and hexagon) and feature (same vs. different) conditions, each of the Experiments 1–3, resulted in a total of 40 stimulus conditions. Each stimulus condition was presented 10 times in random order, thus resulting in a total of 400 trials for each experiment.

**Fig 2:**
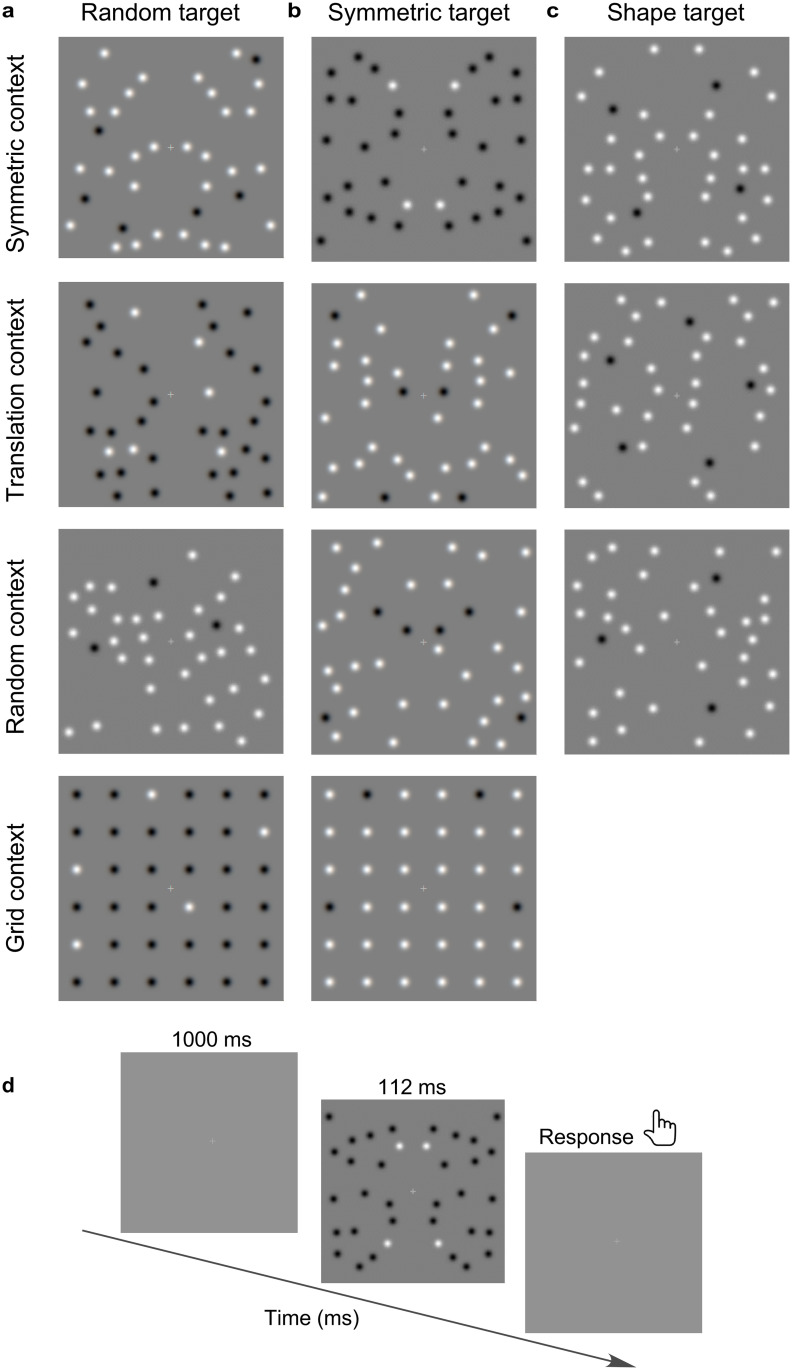
Experiment 4 stimuli. Achromatic dot patterns made of 36 elements that were divided into target (either 3, 4, 5 or 6) and context elements. The target elements were placed either **(a)** random, **(b)** mirror symmetric, or **(c)** on the vertices of simple geometric shapes (equilateral triangle, square, pentagons, hexagon). Context elements were organised either mirror symmetric (top), translation symmetric (2^nd^ row), randomly (3^rd^ row) and in a regular grid (bottom). Note that the combination of shape target—grid context was not used as placing target elements on the vertices of simple shapes (triangle, pentagon, hexagon) within a grid would break the regularity of the grid pattern. **(d)** Schematic representation of the procedure.

In Experiment 4, we examined how spatial organisation of context and target elements modulate target numerosity estimation. Stimuli consisted of dot patterns made of 36 elements (achromatic Gaussian blobs) that were divided into *target* (either 3, 4, 5 or 6) and *context* elements. The target and context elements were dissociated by luminance polarity (black target—white context or white target—black context; Fig 2). We varied the spatial arrangement of (a) *target* elements by placing the elements either random (Fig 2a), mirror symmetric (Fig 2b), on the vertices of simple shapes (equilateral triangle, square, pentagons, hexagon; Fig 2c), and (b) *context elements* by arranging the elements either mirror symmetric, translation symmetric, random or in a regular grid pattern (top to bottom panels in Fig 2). For comparison, we also used a ‘no-context’ (or target only) condition (Fig 1a and 1c). For the shape-target conditions, the orientation of each virtual shape was randomised from trial to trial. We used all target-context combinations, except shape target embedded in a grid context as placing target elements on the vertices of simple shapes within a grid would break the regularity of the grid pattern. Given the number of elements (3, 4, 5, 6), target arrangement (random, symmetric, shape) and context arrangement (mirror symmetric, translation symmetric, random, regular grid, no context), Experiment 4 resulted in 56 conditions. Each target-context condition was presented 8 times in random order, resulting in a total number of 448 trials.

### 2.3. Procedure—Accuracy and reaction times

Each experimental session started with a fixation cross (1000 ms), followed by a stimulus presented for 112 ms (Experiments 1 and 2) or 120 ms (Experiment 3 and 4) and a uniform mid-grey background. In Experiments 1–3, the 400 trials were divided into five blocks of 80 trials each (two repeats for each condition in each block). In Experiment 4, target-context conditions were blocked by the luminance polarity of the target elements, that is, white target—black context or vice-versa. There were four blocks (two for each luminance polarity target-context combination), with each block containing 112 trials (two repeats for each stimulus condition in each block). We used a speeded response task in which the observer had to indicate as quickly and accurately as possible the number of elements perceived on the screen by pressing the corresponding key (e.g., ‘3’ for three elements, ‘4’ for four elements with the middle and index fingers of the left hand respectively, and ‘5’ for five elements and ‘6’ for six elements with the index and middle fingers of the right hand). We asked participants to use both their hands while responding to balance differences in motor responses within and between participants. No feedback was given to observers after responding. In Experiment 4, observers were told the luminance polarity of the target elements (white or black) before each block.

In each experiment, for each stimulus condition and observer, we measured accuracy (proportion correct responses) and overall reaction times (RTs). We then calculated the average across-participants for each of these measures and the standard error. All data were subjected to either three- or two-way repeated-measures ANOVAs carried out separately for each shape configuration (triangle, square, pentagon, hexagon) in Experiments 1–3 or type of target arrangement (symmetric, random, shape) in Experiment 4, with each model explained fully in the *Supporting Information* section (S1 Appendix). Greenhouse-Geisser corrections were used where applicable. To demonstrate the magnitude of effects, partial eta-square ($\eta_{p}^{2}$) is also reported.

## 3. Results

### 3.1. Effect of colour

**Fig 3:**
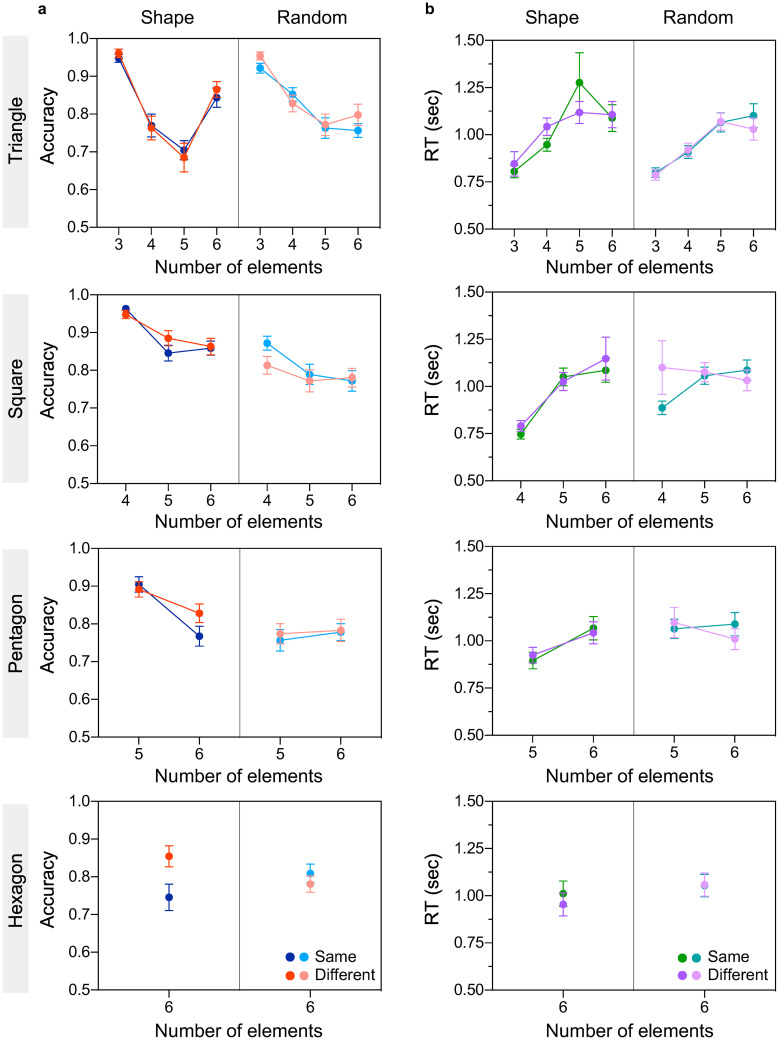
Experiment 1 results (effect of colour). **(a)** Accuracy and **(b)** reaction time are plotted as a function of number of elements for simple shape (left side of each panel) and random (right side of each panel) configurations, and for same (blue/green symbols) and different (red/purple symbols) colour conditions. Top to bottom panels correspond to different simple shape configurations: Equilateral triangle (top panel), square, pentagons and hexagon (bottom panel) configuration. The error bars indicate standard error of the mean (±1 SEM).

Fig 3 shows accuracy (Fig 3a) and reaction time (Fig 3b) as a function of number of elements for shape (left) and random (right) spatial configurations, and for same (blue/green symbols) and different (red/purple symbols) colour conditions. Top to bottom panels correspond to different shape configurations: equilateral triangle (top), square, pentagon, and hexagon (bottom). These results indicate (a) comparable accuracy and comparable RTs between same and different colour conditions (compare light/dark purple with light/dark green symbols); (b) that accuracy decreases and RTs are slower with increasing the number of elements; (c) higher accuracy and faster RTs are obtained when elements were placed on the vertices of simple shapes compared to random (compare light and dark purple/green), and when number of elements matched the number of vertices of each shape (i.e., 3 elements on an equilateral triangle, 4 elements on a square, and so on).

A three-way repeated-measures ANOVA with factors spatial configuration (shape vs. random), number of elements (3, 4, 5, 6), and colour (same vs. different) was carried out for triangle, square and pentagon conditions for accuracy and RT data, separately. *For accuracy data*, the analysis revealed a significant main effect of number of elements (p’s < 0.001 for triangle and square, except for pentagon p = 0.078, $\eta_{p}^{2}=0.067$) suggesting that accuracy decreases as number of elements increases. The effect of spatial configuration was also significant (p < 0.001 for square and pentagon, except for triangle p = 0.174, $\eta_{p}^{2}=0.04$) indicating that accuracy is higher for shape compared to random configurations. However, the effect of colour was not significant for all shape configurations (all p’s > 0.176; see S1 Appendix) suggesting that colour similarity does not improve performance.

In addition, a significant two-way interaction effect between the number of elements and spatial configuration (F(2.67, 119.98) = 19.5, p < 0.001, $\eta_{p}^{2}=0.302$ for triangle and F(1, 45) = 12.61, p < 0.001, $\eta_{p}^{2}=0.219$ for pentagon only) and a three-way interaction effect between the number of elements, spatial configuration, and colour for pentagon only (F(1, 45) = 5.13, p = 0.028, $\eta_{p}^{2}=0.102$) were found. Bonferroni corrected post-hoc analysis revealed that performance for all shape conditions for which the number of elements matched the number of shape vertices (i.e., 3 elements placed on a triangle, 4 elements placed on a square, 5 elements placed on a pentagon) were significantly better from all other number of elements and random conditions, except for 3 elements on triangle condition in which both shape (equilateral triangle) and random (scalene triangle which is also a shape) conditions were not significant. All pairwise comparisons between different number of elements conditions were significant, irrespective of spatial configuration, except between 5 and 6 elements.

For hexagon condition, a two-way repeated-measures ANOVA revealed a significant effect of colour (F(1, 45) = 4.8, p = 0.034, $\eta_{p}^{2}=0.096$), with lower accuracy for different compared to same colour condition. The effect of spatial configuration was not significant (p = 0.835). However, there was a significant interaction effect between colour and spatial configuration (F(1, 45) = 13.73, p < 0.001, $\eta_{p}^{2}=0.234$) which revealed that same and different colour conditions were significant only for shape (p = 0.001) but not for random configurations.

*For RTs data*, the analysis revealed a significant effect of number of elements (p < 0.001 for triangle and square, except for pentagon p = 0.116, $\eta_{p}^{2}=0.056$), suggesting faster RTs for small number of elements. The effect of spatial configuration was also significant (p < 0.01 for triangle and pentagon, but not for square p = 0.057, $\eta_{p}^{2}=0.078$), indicating faster RTs for triangle and pentagon shape compared to random configurations. As with accuracy, the main effect of colour was not significant (all p’s > 0.161), suggesting that colour inconsistency does not affect RTs. In addition, there was also a significant interaction effect between number of elements and spatial configuration for square (F(2, 90) = 6.53, p = 0.002, $\eta_{p}^{2}=0.127$) and pentagon (F(1, 45) = 12.74, p < 0.001, $\eta_{p}^{2}=0.221$) conditions only. All Bonferroni-corrected pairwise comparisons between shape conditions in which the number of elements matched the number of vertices (e.g., 3 elements placed on a triangle; 4 elements placed on a square; 5 elements placed on a pentagon) and all other number of elements and configuration conditions were significant (p < 0.05) except between 5 and 6 elements for the square condition and between 5 random elements and 6 elements for the pentagon condition.

For hexagon condition, the two-way ANOVA revealed a significant main effect of spatial configuration (F(1, 45) = 4.31, p = 0.044, $\eta_{p}^{2}=0.087$) suggesting that RTs are faster in the shape compared to the random condition. However, the main effect of colour and the interaction effect between colour and spatial configuration were not significant (all p’s > 0.34).

Altogether, this experiment reveals that spatial configuration and number of elements but not colour inconsistency affect numerosity estimation within the subitizing range.

### 3.2. Effect of luminance polarity

**Fig 4:**
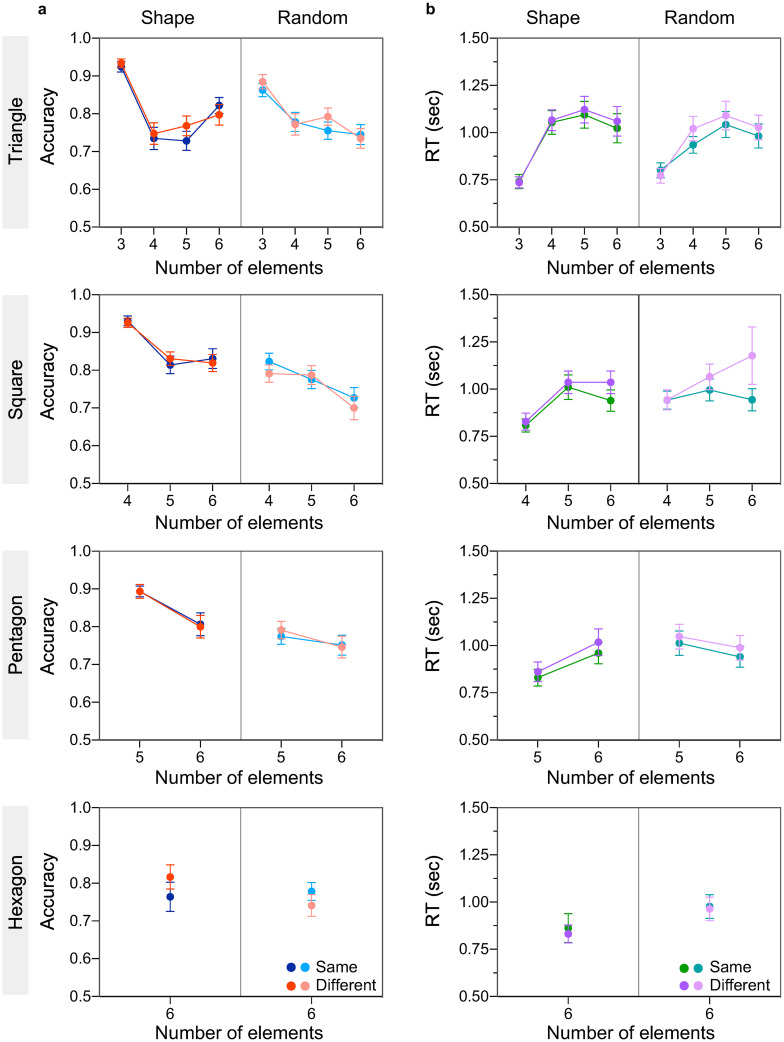
Experiment 2 results (effect of luminance polarity). **(a)** Accuracy and **(b)** reaction time are plotted as a function of number of elements for simple shape (left side of each panel) and random (right side of each panel) configurations, and for same (blue/green symbols) and different (red/purple symbols) luminance poalrity conditions. Top to bottom panels correspond to different simple shape configurations: Equilateral triangle (top panel), square, pentagons and hexagon (bottom panel) configuration. The error bars indicate standard error of the mean (±1 SEM).

Accuracy (Fig 4a) and reaction times (Fig 4b) are shown as a function of number of elements for shape (left side) and random (right side) conditions, for the same (blue/green) and different (red/purple) luminance polarity conditions. Top to bottom panels indicate different shape configurations: equilateral triangle (top), square, pentagon, hexagon (bottom). As with colour, these results indicate (a) comparable accuracy and comparable RTs for same and different luminance polarity conditions; (b) lower accuracies and slower RTs with increasing number of elements; (c) higher accuracy and faster RTs when elements were placed on simple geometric shapes compared to random, and when they matched the number of shape vertices.

A three-way repeated-measures ANOVA with factors number of elements (3,4,5,6), spatial configuration (shape vs. random), and luminance polarity (same vs. different) was carried out for triangle, square and pentagon configurations, and for accuracy and RTs data, separately. The analysis revealed a significant main effect of number of elements and spatial configuration (all p’s < 0.02 for accuracy and RTs), suggesting higher accuracy and faster RTs for shape compared to random configuration, and for smaller than larger number of elements. However, the main effect of luminance polarity was not significant for accuracy (all p’s > 0.218) and RTs (p = 0.06 for triangle, except for square p = 0.012 and pentagon p = 0.018). Bonferroni corrected multiple comparisons for square and pentagon conditions revealed that all t-tests between same and different polarity conditions were *not* significant (all p’s > 0.999).

In addition, significant interaction effects between number of elements and spatial configuration were found for triangle (p < 0.001 for accuracy and RTs), square (p = 0.005 for accuracy only) and pentagon (p < 0.017 for accuracy and RTs) conditions. All Bonferroni corrected pairwise comparisons between shape conditions in which the number of elements matched the number of vertices (i.e., 3 elements placed on a triangle; 4 elements on a square; 5 elements on a pentagon) and all other spatial arrangement and number of elements conditions were significant (all p’s < 0.05), except for 3 elements on triangle conditions in which both shape (equilateral triangle) and random (scalene triangle) were not significant (p’s > 0.999).

For hexagon condition, two-way repeated-measures ANOVA revealed that the effect of luminance polarity was not significant (p > 0.6 for accuracy and RTs; see S1 Appendix), while the effect of spatial configuration was significant for RTs (F(1, 38) = 6.46, p = 0.015, $\eta_{p}^{2}=0.145$) but not accuracy (F(1, 38) = 1.01, p = 0.322, $\eta_{p}^{2}=0.026$). In addition, a significant interaction effect between luminance polarity and spatial configuration was found for accuracy only (F(1, 38) = 8.06, p = 0.007, $\eta_{p}^{2}=0.175$). However, all Bonferroni corrected post-hoc comparisons were not significant (all p’s > 0.112).

In sum, we found that spatial configuration and number of elements but not luminance polarity affect numerosity estimation within the subitizing range.

### 3.3. Effect of orientation

**Fig 5:**
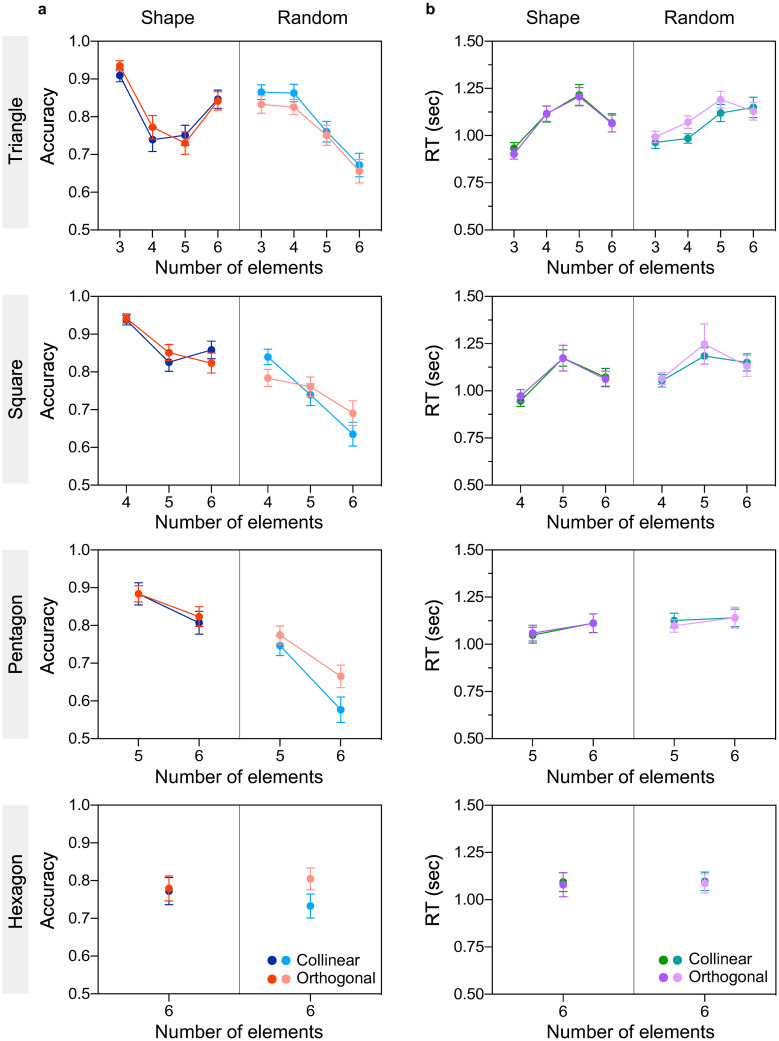
Experiment 3 results (effect of orientation). **(a)** Accuracy and **(b)** reaction time are plotted as a function of number of elements for simple shape (left side of each panel) and random (right side of each panel) configurations, and for colinear (blue/green symbols) and orthogonal (red/purple symbols) to contour-path orientation conditions. Top to bottom panels correspond to different simple shape configurations: Equilateral triangle (top panel), square, pentagons and hexagon (bottom panel) configuration. The error bars indicate standard error of the mean (±1 SEM).

Accuracy (Fig 5a) and reaction times (Fig 5b) are shown as a function of number of elements for shape and random spatial configurations, and for colinear (blue/green) and orthogonal (red/purple) to contour-path orientation conditions. Similar to Experiments 1 and 2, these results show (a) comparable accuracy and comparable RTs between colinear and orthogonal orientation conditions, (b) lower accuracies and slower RTs with increasing number of elements, and (c) higher accuracies and faster RTs for shape compared to random configuration conditions, and when number of elements matches number of vertices.

A three-way repeated-measures ANOVA with factors number of elements (3, 4, 5, 6), spatial configuration (shape vs. random), and orientation (colinear vs. orthogonal) was carried out for triangle, square, and pentagon configurations, and for accuracy and RTs data separately. The analysis revealed a significant main effect of number of elements for accuracy (all p’s < 0.002) and RTs (p < 0.001 for triangle and square except for pentagon p = 0.075, $\eta_{p}^{2}=0.073$) indicating better performance for lower number of elements. The effect of spatial configuration was also significant for accuracy (all p’s < 0.001) and RTs (p < 0.036 for square and pentagon, except for triangle p = 0.812) indicating higher accuracy and faster RTs for shape compared to random configurations. However, the effect of orientation was not significant for accuracy (p > 0.455 for triangle and square but not pentagon F(1, 42) = 5.73, p = 0.021, $\eta_{p}^{2}=0.12$) and RTs (all p’s > 0.088) suggesting comparable performance with colinear and orthogonal orientations. In addition, a significant interaction effect between number of elements and spatial configuration was found for accuracy (p < 0.005 for triangle and square only) and RT for triangle only (F(2.70, 113.6) = 12.21, p < 0.001, $\eta_{p}^{2}=0.225$). There was also a significant interaction between spatial configuration and orientation for triangle configuration (F(1, 42) = 4.58, p = 0.038, $\eta_{p}^{2}=0.098$ for accuracy; F(1, 42) = 12.36, p = 0.001, $\eta_{p}^{2}=0.227$ for RTs) and between number of elements, spatial configuration and orientation for square configuration for accuracy only (F(1.97, 82.69) = 5.33, p = 0.007, $\eta_{p}^{2}=0.113$). All other interaction effects were not significant (p > 0.093). For square and pentagon conditions, Bonferroni corrected post-hoc analysis on accuracy data revealed significant pairwise comparisons between shape conditions in which the number of elements matched the number of vertices (i.e., 4 elements on square vertices; 5 elements on pentagon vertices) and all other number of elements and configuration conditions (p < 0.022). For triangle condition, all pairwise comparisons between 3 elements on both shape and random configurations were significant from all other conditions (p’s < 0.034), for both accuracy and RTs.

For hexagon condition, the two-way repeated-measures ANOVA revealed no significant main effect of orientation and spatial configuration, and no significant interaction effect (all p’s > 0.06 for accuracy and RTs; see S1 Appendix).

Altogether this experiment indicates that spatial configuration and number of elements, but not their local orientation affects numerosity estimation within the subitizing range.

### 3.4. Effect of context

**Fig 6:**
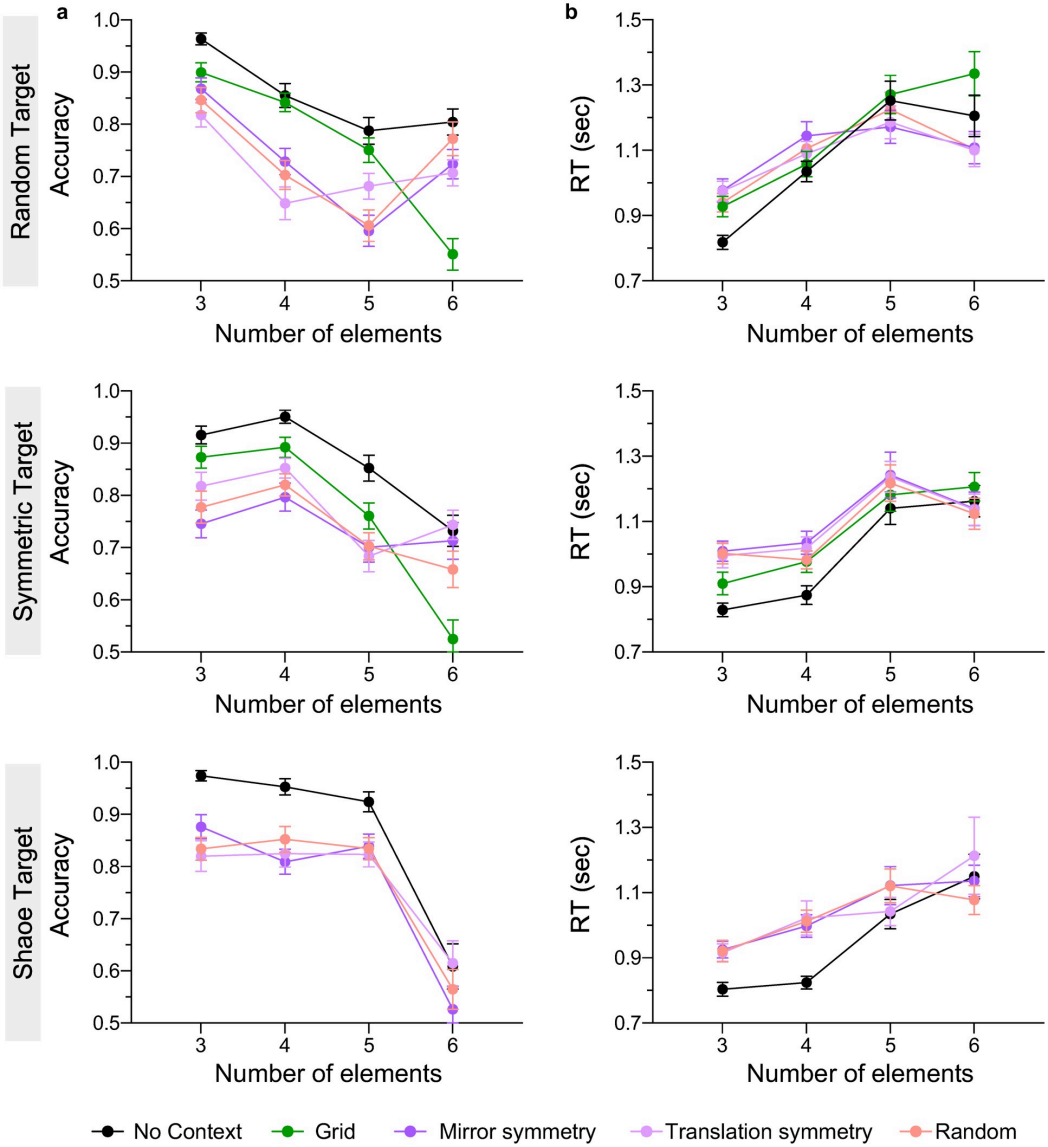
Experiment 4 results (context effects). **(a)** Accuracy and **(b)** reaction time are plotted as a function of number of elements and context configuration (no context, grid, mirror symmetric, translation, random), for different type of target arrangements: Random target (top panels), symmetric target (middle panels) and shape target (bottom panel). The error bars indicate standard error of the mean (±1 SEM).

Fig 6 shows accuracy (Fig 6a) and reaction times (Fig 6b) as a function of number of target elements for different context arrangements and different target configurations (top to bottom: random, symmetric and shape). The results indicate that the presence of context decreases accuracy and increases RTs compared to the ‘no context’ condition. Overall, there was better performance with the grid compared to mirror symmetric, translation symmetric and random contexts, except for six target-element condition in which the grid context yielded the lowest accuracy. Random, translation and mirror symmetric contexts produced comparable accuracy and comparable RTs, while shape target configurations resulted in overall higher accuracy compared to random and symmetric targets. Finally, accuracy was higher and RTs faster for small (3,4) compared to large (5,6) number of elements.

A two-way repeated-measures ANOVAs with factors context type (random, grid, mirror symmetric, translation symmetric, no context) and number of elements (3,4,5,6) was carried out separately for each target type (random, symmetric, shape) on the accuracy and RT data, respectively. The effect of context type was significant for all target configurations for accuracy and RTs (all p’s < 0.001; see S1 Appendix) indicating that spatial organisation of context affects target numerosity estimation. The effect of number of elements was also significant for accuracy and RTs (all p’s < 0.001) suggesting that increasing number of elements reduces accuracy and increases RTs.

Finally, a significant interaction effect between number of elements and context type was found for all target configurations for both accuracy (all p’s < 0.004) and RTs (all p’s < 0.001 except for shape configuration p = 0.075; $\eta_{p}^{2}=0.054$). For accuracy data, all Bonferroni corrected pairwise comparisons between the ‘no context’ and random, mirror symmetric, translation conditions were significant (all p’s < 0.023 except for 6 elements conditions p’s > 0.07), indicating higher accuracy for no context compared to random and symmetric contexts. The pairwise comparisons between the grid and ‘no context’ condition were also significant for 3 and 6 elements conditions only (p’s < 0.015). In addition, all pairwise comparisons between mirror symmetric, translation and random contexts were not significant (all p’s > 0.07), except for 6 elements condition, in which the accuracy for the grid context condition was significantly lower than all other context types (all p’s < 0.01). For reaction time data, the fast RTs were obtained with no context condition, mainly for 3 and 4 elements (all p’s < 0.001). RTs obtained with symmetric targets were significantly slower for 5 compared to 4 elements (all p’s < 0.01), while for random and shape targets RTs increased gradually with the number of elements from 3 to 5 elements (all p’s < 0.045), but not from 5 to 6.

As for *target spatial arrangement*, shape target produced highest accuracy and fastest RTs, followed by symmetric and random targets. To examine the effect of target arrangement, we carried out two-way repeated-measures ANOVAs with factor target type (shape, symmetric, random) and number of elements (3, 4, 5, 6), separately for each context type. The analysis showed a significant effect of number of elements (all p’s < 0.001 for accuracy and RTs) and target type for RTs only (all p’s < 0.005 except for translation symmetric context F(1.27, 58.26) = 1.82, p = 0.182; $\eta_{p}^{2}=0.038$). For accuracy, the effect of target organisation was significant for the random and translation symmetric contexts only (all p’s < 0.026) but not for grid (p = 0.883), mirror symmetric (p = 0.104) and no context (p = 0.675) conditions (see S1 Appendix). However, a significant interaction effect between the number of elements and target type was found for all context types (p < 0.001) except grid context (p > 0.158 for accuracy and RTs). Bonferroni corrected multiple comparison analysis for accuracy data showed that all pairwise comparisons were significant (all p’s < 0.03) except between random and shape target arrangements for 3 elements (i.e., equilateral vs. scalene triangle), between random and symmetric target for 4 and 6 elements embedded in symmetric context, and between shape and symmetric target embedded in random context (all p’s > 0.01).

## 4. Discussion

We have examined whether numerosity mechanisms around the subitizing range are selective to colour, luminance polarity and orientation of elements, and determined whether these selectivities are inherited from feature-selective shape channels. We also examined whether spatial organisation of context and target elements modulates target numerosity perception. Our results show (a) comparable performance with stimuli defined along the same and different feature dimension suggesting that mechanisms involved in numerosity estimation around the subitizing range are not selective to colour, luminance polarity and orientation; (b) higher accuracy and faster RTs for shape compared to random configurations, suggesting that numerosity estimation around the subitizing range is facilitated by a shape-template matching mechanism that is not selective to these features; (c) reduced performance for all target spatial organisations when presented in context than in isolation, with increasing target numbers leading to overall reduced accuracy and longer RTs; (d) significantly better performance with the grid compared to mirror symmetric, translation symmetric and random contexts (except for 6 target-elements condition where the grid context yielded the lowest accuracy) and for shape compared to symmetric and random target arrangements. These results suggest that symmetric, translation and random context organisations inhibit target-numerosity coding stronger than regular/grid context.

Our findings from Experiments 1–3 showing improved performance (higher accuracy and faster RTs) for shape compared to random configurations and also when the number of elements matched the number of shape vertices (i.e., 3 elements on a triangle compared to 4, 5, or 6 elements on a triangle) provide additional support for the idea that numerosity estimation around the subitizing range is facilitated by a shape-template matching mechanism which takes into account the relationship between points of maximum curvature or vertices [26]. Further support for this idea comes from a recent study showing that sensitivity to visual form (static Glass patterns) correlated with numerical abilities in individuals with developmental dyscalculia [30]. One might note that in Experiment 1 (colour), the effect of shape configuration (shape vs. random) for the triangle condition only was not found statistically significant. One reason for this is that the shape condition corresponded always to an equilateral triangle, while a shape randomly-sampled by 3 elements will be perceived as a scalene triangular shape. Irrespective of such instances, the effect of spatial configuration was found in all other conditions and experiments.

Across the three experiments we also found that mechanisms involved in numerosity estimation around the subitizing range are not selective to colour, luminance polarity and orientation, and this lack of selectivity occurred irrespective of spatial configuration (shape vs random) of elements. Given that numerosity estimation around the subitizing range is facilitated by a shape-template matching process which takes into consideration the spatial relationship between points of maximum curvature (corners or vertices) and that an inconsistency in non-shape attributes (luminance polarity, colour, orientation) affect shape perception, then why numerosity estimation around subitizing range was found to be invariant to changes in these features? Shape processing studies showed that the positions and non-shape attributes of local elements are first combined into a hierarchy of intermediate shape features such as curves and parts of shape that are luminance-polarity [32,34,35], colour [37,38] and orientation [31,39] selective, and these are further combined in ways that are gradually more invariant to changes in these local attributes, therefore making the higher stages of shape processing invariant to local changes in these attributes [72]. Studies that examined grouping of spatially separated collinear line segments have shown that grouping phenomena involving long-range neural interactions (i.e., interactions between segments or signals located in small regions that may be separated by large distances [73]) are not sensitive to changes in luminance polarity [74,75] and color [76,77], while those mediated by short-range interactions are disrupted by changes in luminance polarity and colour. Thus, the mechanisms involved in numerosity estimation around the subitizing range follow higher stages of shape processing that are agnostic to changes in colour, luminance polarity and orientation, and are likely mediated by long-range neural interactions.

The non-selectivity to orientation of subitizing mechanisms found in our Experiment 3 is in keeping with findings from adaptation studies showing that numerosity estimation above the subitizing range are non-selective to orientation [48]. On the other hand, these findings differ from DeWind et al [47] study showing that aligning the orientations of elements increases perceived numerosity while increasing orientation variance decreased their perceived numerosity. However, low density textures (as used in [47]) have been found to be discriminated on the basis of orientation variance [78–80] without the need for explicit coding of *individual positions* of elements. Thus, simple orientation statistics of textures could drive a range of numerosity estimation tasks (as in [47]) and/or texture tasks.

Our finding that subitizing mechanisms are non-selective to colour might seem at first sight at odds with Gross et al. [49] finding that numerosity estimation *above* the subitizing range (12–48 elements) is colour selective only (but *not* orientation-selective [48]). The most likely reason for this discrepancy is that in Gross et al. [49] the adaptor and test patterns differed not only in numerosity but also in elements’ density, and therefore, the sense of number and density were intertwined [50–52]. Given that texture-density mechanisms are colour selective [53,54], it is likely that the selectivity to colour found in [49] is due to texture-density rather than numerosity mechanisms.

The finding that subitizing mechanisms are non-selective to colour and luminance polarity complement a number of previous findings exploring the role of these features in shape processing [81,82], texture-processing [83,84] and in a variety of figure-ground relationships [85–92], which all failed to find evidence for colour-specific and luminance-polarity (‘on-off’) specific channels mediating the specific dimension of interest (e.g., global shapes/lines, global motion, texture-shape, stereoscopic depth). The finding that subitizing mechanisms do not depend on ‘on-off’ luminance-polarity, colour or orientation channel interactions has implications for studies investigating groupitizing phenomena which are thought to rely on the recruitment of the subitizing system [55,56,60–63]. These groupitizing studies showed that when arrays of elements are grouped into small clusters of no more than 5–6 elements of the same colour (often forming arrays/lines or simple geometric shapes), numerosity estimation is more rapid and accurate compared to when the clusters were of different colours (but see [58] showing that colour grouping reduces perceived numerosity). Given the *non-selectivity* to colour of the subitizing system, it is likely that any improvement in performance found in these groupitizing studies is likely mediated by feature-based attention mechanisms that facilitate grouping (increase stimulus saliency) instead of the recruitment of the subitizing system. It is worth noting that groupitizing studies showing improved performance with colour-grouped displays have mainly used stimuli made of 2 (black/white) or 3 colours [55,56,61], while those that use stimuli containing 6 or more colours reported poorer performance [58]. These differences in groupitizing performance suggest that the number of colours in the stimuli also affects numerosity estimation. Using stimuli made of different numbers of spatially overlapping dots of many colors, Halberda et al. [93] showed that observers can select at a glance, group and estimate numerosities on the basis of shared colour for approximately three color subsets (as individual sets), and this limit was comparable to the three-item limit of parallel attention. In our Experiment 1, the same-colour condition always comprised elements of one colour, while different-colour condition (Fig 1b) contained between 3 to 6 colours, which is above the three-item/colours limit of parallel attention [93]. Hence, it is unlikely that the number of colours/ three-item limit of parallel attention contributed to the lack of colour selectivity found in Experiment 1.

Feature-based attention mechanisms have been found to mediate many visual processes [94]. Several studies have shown that attention-to-colour (and/or luminance polarity) improves performance for symmetry detection [95], global motion discrimination [86,90,96], stereoscopic segmentation [91] in the absence of colour or luminance-polarity selectivity of these mechanisms (symmetry, motion, disparity). Furthermore, evidence showing decreased performance with increasing number of colours in the stimuli has been also reported for symmetry detection [95,97] *with* or *without* attention to colour. In a similar vein, event-related potential studies of numerosity estimation investigating the ‘individuation’ of a group of target elements from homogeneous distractor elements via colour have identified N2pc and CDA (Contralateral Delayed Activity) [66] components that are elicited only under lateralized stimulus presentations, and likely linked to feature-based attention mechanisms. Using target only elements (no context) that had either same (all white or all black) or different luminance polarity (white and black), Gheorghiu and Dering [26] showed that the Left Mid-Frontal (LMF) component was agnostic to the luminance polarity of elements and specifically encoded numerosity (by separating low and high numbers of elements), irrespective of their spatial configuration, while the N2 component was differentially modulated by same and different luminance polarity, and was explicitly linked to stimulus spatial configuration.

Finally, in Experiment 4 we found that symmetric, translation and random organisations of context elements inhibit the encoding of low-numerosity targets stronger than regular/grid contexts, with this pattern being reversed for the highest numerosity (6 elements). While overall, we find better performance for shape compared to symmetric and random targets made of 3–5 elements for all context types, for 6 elements condition accuracy declines more prominently for shape compared to symmetric and random targets (compare top to bottom panels in Fig 6). This suggests that context-target interaction effects in numerosity estimation (and the lower performance for grid for 6 elements condition) depend on target structure. Although some studies showed that mirror symmetric patterns were perceived as less numerous than random patterns [42], our results suggest that mirror symmetric, translation and random context organisations affect target numerosity in a comparable way.

## Supporting information

S1 AppendixOutput of ANOVAs.Accuracy and reaction time data were subjected to either three- or two-way repeated-measures ANOVAs carried out separately for each shape configuration (triangle, square, pentagon, hexagon) in Experiments 1–3 or type of target arrangement (symmetric, random, shape) in Experiment 4. The output of each model (ANOVA table) is provided together with the Greenhouse-Geisser correction and partial eta-square ($\eta_{p}^{2}$).(PDF)
