# Supplementary material for: Spatial and chromatic properties of numerosity estimation in isolation and context
Source: PLoS One. 2022 Sep 15;17(9):e0274564. doi: 10.1371/journal.pone.0274564 (PMC9477322; doi:10.1371/journal.pone.0274564)
Supplement: S1 Appendix — Accuracy and reaction time data were subjected to either three- or two-way repeated-measures ANOVAs carried out separately for each shape configuration (triangle, square, pentagon, hexagon) in Experiments 1–3 or type of target arrangement (symmetric, random, shape) in Experiment 4. The output of each model (ANOVA table) is provided together with the Greenhouse-Geisser correction and partial eta-square (ηp2). (PDF) [file pone.0274564.s001.pdf]

Supporting Information - S1 Appendix

Spatial and chromatic properties of numerosity estimation in isolation and context

Elena Gheorghiu[1,✉] & Dirk Goldschmitt [1]

[1] *University of Stirling, Department of Psychology, Stirling, FK9 4LA, Scotland, United Kingdom*

✉Corresponding author: elena.gheorghiu@stir.ac.uk

Accuracy and reaction time data were subjected to either three- or two-way repeated-measures ANOVAs carried out separately for each shape configuration (triangle, square, pentagon, hexagon) in Experiments 1-3 or type of target arrangement (symmetric, random, shape) in Experiment 4. The output of each model is detailed in the tables below. Greenhouse-Geisser corrections were used where applicable. To demonstrate the magnitude of effects, partial eta-square (η_p^2) is also reported.

1. Experiment 1: *Effect of Colour*

1.1. Accuracy data

Triangle

**Source of variation**	**df_n, df_d**	**MSE**	**F**	**p-value**	**η_p^2**
Number of elements	2.38, 106.96	0.06	33.48	< 0.001	0.427
Spatial configuration	1, 45	0.02	1.85	0.18	0.04
Colour	1, 45	0.01	1.08	0.304	0.023
Number of elements x Spatial configuration	2.67, 119.98	0.01	19.5	< 0.001	0.302
Number of elements x Colour	2.65, 119.22	0.01	1.8	0.158	0.038
Spatial configuration x Colour	1, 45	0.01	0.56	0.457	0.012
Number of elements x Spatial configuration x Colour	2.31, 104.08	0.02	0.4	0.701	0.009

Square

**Source of variation**	**df_n, df_d**	**MSE**	**F**	**p-value**	**η_p^2**
Number of elements	1.44, 64.61	0.04	11.9	< 0.001	0.209
Spatial configuration	1, 45	0.02	59.99	< 0.001	0.571
Colour	1, 45	0.01	0.68	0.413	0.015
Number of elements x Spatial configuration	1.70, 76.32	0.02	0.64	0.505	0.014
Number of elements x Colour	1.70, 76.46	0.02	2.33	0.112	0.049
Spatial configuration x Colour	1, 45	0.01	2.42	0.127	0.051
Number of elements x Spatial configuration x Colour	1.80, 80.78	0.01	1.28	0.281	0.028

Pentagon

**Source of variation**	**df_n, df_d**	**MSE**	**F**	**p-value**	**η_p^2**
Number of elements	1, 45	0.05	3.25	0.078	0.067
Spatial configuration	1, 45	0.02	27.97	< 0.001	0.383
Colour	1, 45	0.01	1.89	0.176	0.04
Number of elements x Spatial configuration	1, 45	0.02	12.61	< 0.001	0.219
Number of elements x Colour	1, 45	0.02	1.22	0.275	0.026
Spatial configuration x Colour	1, 45	0.01	0.4	0.528	0.009
Number of elements x Spatial configuration x Colour	1, 45	0.01	5.13	0.028	0.102

Hexagon

**Source of variation**	**df$_n$, df$_d$**	**MSE**	**F**	**p-value**	η_p^2
Spatial configuration	1, 45	0.001	0.044	0.835	< 0.001
Colour	1, 45	0.074	4.8	0.034	0.096
Spatial configuration x Colour	1, 45	0.215	13.73	< 0.001	0.234

1.2. Reaction time data

Triangle

**Source of variation**	**df$_n$, df$_d$**	**MSE**	**F**	**p-value**	η_p^2
Number of elements	1.82, 82.03	0.29	21.6	< 0.001	0.324
Spatial configuration	1, 45	0.1	8.35	0.006	0.157
Colour	1, 45	0.08	0.19	0.667	0.004
Number of elements x Spatial configuration	1.82, 81.87	0.15	1.1	0.332	0.024
Number of elements x Colour	1.93, 86.66	0.12	1.89	0.159	0.04
Spatial configuration x Colour	1, 45	0.09	0.13	0.721	0.003
Number of elements x Spatial configuration x Colour	2.10, 94.64	0.1	2.39	0.094	0.051

Square

**Source of variation**	**df$_n$, df$_d$**	**MSE**	**F**	**p-value**	η_p^2
Number of elements	1.48, 66.62	0.27	11.14	< 0.001	0.198
Spatial configuration	1, 45	0.15	3.81	0.057	0.078
Colour	1, 45	0.13	2.03	0.161	0.043
Number of elements x Spatial configuration	1.71, 77.03	0.17	6.53	0.004	0.127
Number of elements x Colour	1.65, 74.16	0.15	2.06	0.143	0.044
Spatial configuration x Colour	1, 45	0.14	0.27	0.603	0.006
Number of elements x Spatial configuration x Colour	1.60, 71.98	0.14	2.1	0.139	0.045

Pentagon

**Source of variation**	**df$_n$, df$_d$**	**MSE**	**F**	**p-value**	η_p^2
Number of elements	1, 45	0.11	2.69	0.108	0.056
Spatial configuration	1, 45	0.05	12.36	0.001	0.215
Colour	1, 45	0.05	0.2	0.657	0.004
Number of elements x Spatial configuration	1, 45	0.06	12.74	< 0.001	0.221
Number of elements x Colour	1, 45	0.05	3.04	0.088	0.063
Spatial configuration x Colour	1, 45	0.04	0.33	0.566	0.007
Number of elements x Spatial configuration x Colour	1, 45	0.07	0.3	0.584	0.007

Hexagon

**Source of variation**	**df$_n$, df$_d$**	**MSE**	**F**	**p-value**	η_p^2
Spatial configuration	1, 45	0.06	4.31	0.044	0.087
Colour	1, 45	0.06	0.56	0.46	0.012
Spatial configuration x Colour	1, 45	0.05	0.93	0.341	0.02

2. Experiment 2: *Effect of Luminance Polarity*

2.1. Accuracy data

Triangle

**Source of variation**	**df_n, df_d**	**MSE**	**F**	**p-value**	η_p^2
Number of elements	2.09, 79.59	0.06	18.94	< 0.001	0.333
Spatial configuration	1, 38	0.01	5.21	0.028	0.121
Polarity	1, 38	0.01	1.65	0.206	0.042
Number of elements x Spatial configuration	2.80, 106.57	0.01	11.07	< 0.001	0.226
Number of elements x Polarity	2.78, 105.81	0.01	2.56	0.064	0.063
Spatial configuration x Polarity	1, 38	0.01	0.01	0.918	<.001
Number of elements x Spatial configuration x Polarity	2.82, 107.25	0.01	0.27	0.837	0.007

Square

**Source of variation**	**df_n, df_d**	**MSE**	**F**	**p-value**	η_p^2
Number of elements	1.56, 59.37	0.04	13.07	< 0.001	0.256
Spatial configuration	1, 38	0.01	91.11	< 0.001	0.706
Polarity	1, 38	0	1.57	0.218	0.04
Number of elements x Spatial configuration	1.94, 73.75	0.01	5.76	0.005	0.132
Number of elements x Polarity	1.86, 70.67	0.01	1.83	0.171	0.046
Spatial configuration x Polarity	1, 38	0.01	0.88	0.355	0.023
Number of elements x Spatial configuration x Polarity	1.69, 64.29	0.01	0.12	0.851	0.003

Pentagon

**Source of variation**	**df_n, df_d**	**MSE**	**F**	**p-value**	η_p^2
Number of elements	1, 38	0.06	5.47	0.025	0.126
Spatial configuration	1, 38	0.02	33.86	< 0.001	0.471
Polarity	1, 38	0.01	0.02	0.887	<.001
Number of elements x Spatial configuration	1, 38	0.01	6.19	0.017	0.14
Number of elements x Polarity	1, 38	0.01	0.33	0.569	0.009
Spatial configuration x Polarity	1, 38	0.01	0.26	0.613	0.007
Number of elements x Spatial configuration x Polarity	1, 38	0.01	0.1	0.753	0.003

Hexagon

**Source of variation**	**df_n, df_d**	**MSE**	**F**	**p-value**	η_p^2
Spatial configuration	1, 38	0.04	1.01	0.322	0.026
Polarity	1, 38	0.01	0.22	0.643	0.006
Spatial configuration x Polarity	1, 38	0.01	8.06	0.007	0.175

2.2. Reaction time data

Triangle

**Source of variation**	**df_n, df_d**	**MSE**	**F**	**p-value**	η_p^2
Number of elements	1.69, 64.30	0.18	31.19	< 0.001	0.451
Spatial configuration	1, 38	0.02	8.09	0.007	0.175
Polarity	1, 38	0.03	3.57	0.066	0.086
Number of elements x	2.45, 93.17	0.02	6.33	0.001	0.143

Spatial configuration					
Number of elements x Polarity	2.29, 87.05	0.03	1.7	0.184	0.043
Spatial configuration x Polarity	1, 38	0.02	0.66	0.423	0.017
Number of elements x Spatial configuration x Polarity	2.64, 100.37	0.02	0.79	0.49	0.02

Square

**Source of variation**	**df_n, df_d**	**MSE**	**F**	**p-value**	η_p^2
Number of elements	1.70, 64.70	0.13	9.92	< 0.001	0.207
Spatial configuration	1, 38	0.08	6.94	0.012	0.154
Polarity	1, 38	0.09	6.99	0.012	0.155
Number of elements x Spatial configuration	1.41, 53.43	0.12	1.67	0.204	0.042
Number of elements x Polarity	1.10, 41.73	0.15	3.1	0.082	0.075
Spatial configuration x Polarity	1, 38	0.09	1.02	0.319	0.026
Number of elements x Spatial configuration x Polarity	1.19, 45.32	0.14	0.78	0.403	0.02

Pentagon

**Source of variation**	**df_n, df_d**	**MSE**	**F**	**p-value**	η_p^2
Number of elements	1, 38	0.03	4.33	0.044	0.102
Spatial configuration	1, 38	0.04	12.13	0.001	0.242
Polarity	1, 38	0.02	6.08	0.018	0.138
Number of elements x Spatial configuration	1, 38	0.05	18.86	< 0.001	0.332
Number of elements x Polarity	1, 38	0.02	0.41	0.524	0.011
Spatial configuration x Polarity	1, 38	0.01	0.01	0.907	<.001
Number of elements x Spatial configuration x Polarity	1, 38	0.01	0.07	0.795	0.002

Hexagon

**Source of variation**	**df_n, df_d**	**MSE**	**F**	**p-value**	η_p^2
Spatial configuration	1, 38	0.09	6.46	0.015	0.145
Polarity	1, 38	0.06	0.29	0.596	0.007
Spatial configuration x Polarity	1, 38	0.07	0.05	0.83	0.001

3. Experiment 3: *Effect of Orientation*

3.1. Accuracy data

Triangle

**Source of variation**	**df_n, df_d**	**MSE**	**F**	**p-value**	η_p^2
Number of elements	2.02, 85.04	0.08	13.19	< 0.001	0.239
Spatial configuration	1, 42	0.01	18.81	< 0.001	0.309
Orientation	1, 42	0.02	0.57	0.455	0.013
Number of elements x Spatial configuration	2.32, 97.54	0.02	29.99	< 0.001	0.417
Number of elements x Orientation	2.94, 123.38	0.01	0.14	0.934	0.003
Spatial configuration x Orientation	1, 42	0.01	4.58	0.038	0.098
Number of elements x Spatial configuration x Orientation	2.57, 107.78	0.02	1.17	0.322	0.027

Square

**Source of variation**	**df$_n$, df$_d$**	**MSE**	**F**	**p-value**	η_p^2
Number of elements	1.39, 58.44	0.08	12.72	< 0.001	0.232
Spatial configuration	1, 42	0.01	183.29	< 0.001	0.814
Orientation	1, 42	0.01	0.1	0.756	0.002
Number of elements x Spatial configuration	2.00, 83.91	0.02	5.54	0.005	0.117
Number of elements x Orientation	1.98, 83.36	0.01	2.44	0.094	0.055
Spatial configuration x Orientation	1, 42	0.01	0.17	0.686	0.004
Number of elements x Spatial configuration x Orientation	1.97, 82.69	0.01	5.33	0.007	0.113

Pentagon

**Source of variation**	**df$_n$, df$_d$**	**MSE**	**F**	**p-value**	η_p^2
Number of elements	1, 42	0.08	11.04	0.002	0.208
Spatial configuration	1, 42	0.02	94.81	<.001	0.693
Orientation	1, 42	0.02	5.73	0.021	0.12
Number of elements x Spatial configuration	1, 42	0.03	3.89	0.055	0.085
Number of elements x Orientation	1, 42	0.02	2.06	0.158	0.047
Spatial configuration x Orientation	1, 42	0.02	3.26	0.078	0.072
Number of elements x Spatial configuration x Orientation	1, 42	0.01	0.77	0.387	0.018

Hexagon

**Source of variation**	**df$_n$, df$_d$**	**MSE**	**F**	**p-value**	η_p^2
Spatial configuration	1, 42	0.05	0.04	0.839	<.001
Orientation	1, 42	0.02	3.73	0.06	0.081
Spatial configuration x Orientation	1, 42	0.01	3.3	0.076	0.073

***3.2.* Reaction time data**

Triangle

**Source of variation**	**df$_n$, df$_d$**	**MSE**	**F**	**p-value**	η_p^2
Number of elements	2.18, 91.58	0.1	23.19	< 0.001	0.356
Spatial configuration	1, 42	0.01	0.06	0.812	0.001
Orientation	1, 42	0.01	3.04	0.088	0.068
Number of elements x Spatial configuration	2.70, 113.60	0.03	12.21	<.001	0.225
Number of elements x Orientation	2.41, 101.18	0.02	1.85	0.155	0.042
Spatial configuration x Orientation	1, 42	0.01	12.36	0.001	0.227
Number of elements x Spatial configuration x Orientation	2.70, 113.51	0.02	1.74	0.168	0.04

Square

**Source of variation**	**df$_n$, df$_d$**	**MSE**	**F**	**p-value**	η_p^2
Number of elements	1.89, 79.48	0.1	15.74	<.001	0.273
Spatial configuration	1, 42	0.06	11.14	0.002	0.21
Orientation	1, 42	0.07	0.26	0.616	0.006
Number of elements x Spatial configuration	1.22, 51.32	0.09	0.61	0.468	0.014
Number of elements x Orientation	1.30, 54.44	0.08	0.43	0.568	0.01
Spatial configuration x Orientation	1, 42	0.07	0.08	0.781	0.002
Number of elements x Spatial configuration x Orientation	1.33, 55.95	0.11	0.29	0.659	0.007

Pentagon

**Source of variation**	**df_n, df_d**	**MSE**	**F**	**p-value**	η_p^2
Number of elements	1, 42	0.05	3.32	0.075	0.073
Spatial configuration	1, 42	0.03	4.68	0.036	0.1
Orientation	1, 42	0.01	0.2	0.655	0.005
Number of elements x Spatial configuration	1, 42	0.05	0.35	0.558	0.008
Number of elements x Orientation	1, 42	0.02	0.11	0.741	0.003
Spatial configuration x Orientation	1, 42	0.02	0.34	0.563	0.008
Number of elements x Spatial configuration x Orientation	1, 42	0.02	0.56	0.457	0.013

Hexagon

**Source of variation**	**df_n, df_d**	**MSE**	**F**	**p-value**	η_p^2
Spatial configuration	1, 42	0.04	0.03	0.86	<.001
Orientation	1, 42	0.02	0.29	0.591	0.007
Spatial configuration x Orientation	1, 42	0.02	0.01	0.932	<.001

4. Experiment 4: *Effect of Context type*

4.1. Accuracy data

Random target

**Source of variation**	**df_n, df_d**	**MSE**	**F**	**p-value**	η_p^2
Context type	3.18, 146.32	0.03	26.17	<.001	0.363
Number of elements	2.03, 93.61	0.06	42.57	<.001	0.481
Context type x Number of elements	9.24, 425.10	0.03	11.82	<.001	0.204

Symmetric target

**Source of variation**	**df_n, df_d**	**MSE**	**F**	**p-value**	η_p^2
Context type	3.42, 157.37	0.03	18.56	<.001	0.287
Number of elements	2.12, 97.62	0.07	36.3	<.001	0.441
Context type x Number of elements	9.11, 419.22	0.03	8.38	<.001	0.154

Shape target

**Source of variation**	**df_n, df_d**	**MSE**	**F**	**p-value**	η_p^2
Context type	2.83, 130.09	0.02	24.51	<.001	0.348
Number of elements	1.45, 66.69	0.16	49.51	<.001	0.518
Context type x Number of elements	5.84, 268.71	0.03	3.3	0.004	0.067

4.2. Reaction time data

Random target

**Source of variation**	**df_n, df_d**	**MSE**	**F**	**p-value**	η_p^2
Context type	3.40, 156.43	0.03	5.63	< 0.001	0.109
Number of elements	1.74, 79.81	0.17	38.6	< 0.001	0.456
Context type x Number of elements	7.31, 336.47	0.04	9.70	< 0.001	0.175

Symmetric target

Source of variation	df_n, df_d	MSE	F	p-value	η_p^2
Context type	3.02, 139.01	0.03	13.43	< 0.001	0.226
Number of elements	1.78, 81.99	0.13	49.49	< 0.001	0.518
Context type x Number of elements	6.70, 308.23	0.05	3.58	0.001	0.072

Shape target

Source of variation	df_n, df_d	MSE	F	p-value	η_p^2
Context type	1.85, 85.24	0.08	7.84	0.001	0.146
Number of elements	2.02, 93.08	0.18	20.21	< 0.001	0.305
Context type x Number of elements	2.15, 98.77	0.24	2.61	0.075	0.054

Experiment 4: *Effect of Target type*

4.3. Accuracy data

Random context

Source of variation	df_n, df_d	MSE	F	p-value	η_p^2
Target type	1.98, 91.07	0.02	3.91	0.026	0.077
Number of elements	2.10, 96.65	0.06	17.06	< 0.001	0.271
Target type x Number of elements	4.88, 224.53	0.03	19.21	<.001	0.295

Symmetric context

Source of variation	df_n, df_d	MSE	F	p-value	η_p^2
Target type	1.92, 88.21	0.02	2.33	0.106	0.048
Number of elements	2.08, 95.63	0.06	21.5	<.001	0.319
Target type x Number of elements	4.39, 201.87	0.03	20.23	<.001	0.305

Translation symmetric context

Source of variation	df_n, df_d	MSE	F	p-value	η_p^2
Target type	1.98, 91.03	0.02	8.64	<.001	0.158
Number of elements	2.32, 106.80	0.05	10.67	<.001	0.188
Target type x Number of elements	4.63, 213.14	0.03	12.8	<.001	0.218

Grid context

Source of variation	df_n, df_d	MSE	F	p-value	η_p^2
Target type	1, 46	0.02	0.02	0.884	<.001
Number of elements	2.24, 103.25	0.04	87.94	<.001	0.657
Target type x Number of elements	2.33, 107.02	0.03	1.53	0.219	0.032

No context

Source of variation	df_n, df_d	MSE	F	p-value	η_p^2
Target type	1.94, 89.12	0.02	0.38	0.675	0.008
Number of elements	1.93, 88.95	0.04	55.46	<.001	0.547
Target type x Number of elements	3.60, 165.43	0.03	15.95	<.001	0.258

4.4. Reaction time data

Random context

Source of variation	df_n, df_d	MSE	F	p-value	η_p^2
Target type	1.89, 87.05	0.02	8.53	<.001	0.156
Number of elements	1.84, 84.65	0.09	24.08	<.001	0.344
Target type x Number of elements	4.43, 203.70	0.03	4.57	<.001	0.09

Symmetric context

**Source of variation**	**df_n, df_d**	**MSE**	**F**	**p-value**	**η_p^2**
Target type	1.99, 91.74	0.04	5.56	0.005	0.108
Number of elements	2.03, 93.60	0.09	18.47	<.001	0.287
Target type x Number of elements	3.41, 156.66	0.05	3.89	0.008	0.078

Translation symmetric context

**Source of variation**	**df_n, df_d**	**MSE**	**F**	**p-value**	**η_p^2**
Target type	1.27, 58.26	0.11	1.82	0.182	0.038
Number of elements	1.81, 83.32	0.18	10.85	<.001	0.191
Target type x Number of elements	1.96, 90.33	0.21	3.28	0.043	0.067

Grid context

**Source of variation**	**df_n, df_d**	**MSE**	**F**	**p-value**	**η_p^2**
Target type	1, 46	0.03	22.87	<.001	0.332
Number of elements	2.03, 93.59	0.09	45.41	<.001	0.497
Target type x Number of elements	2.65, 121.82	0.03	1.79	0.159	0.038

No context

**Source of variation**	**df_n, df_d**	**MSE**	**F**	**p-value**	**η_p^2**
Target type	1.91, 87.89	0.02	31.68	<.001	0.408
Number of elements	1.61, 74.20	0.15	51.76	<.001	0.529
Target type x Number of elements	4.07, 187.08	0.03	7.05	<.001	0.133
